# Safety Monitoring of COVID-19 mRNA Vaccine First Booster Doses Among Persons Aged ≥12 Years with Presumed Immunocompromise Status — United States, January 12, 2022–March 28, 2022

**DOI:** 10.15585/mmwr.mm7128a3

**Published:** 2022-07-15

**Authors:** Anne M. Hause, James Baggs, Paige Marquez, Winston E. Abara, Jane Gwira Baumblatt, Deborah Thompson, John R. Su, Tanya R. Myers, Julianne Gee, Tom T. Shimabukuro, David K. Shay

**Affiliations:** ^1^CDC COVID-19 Emergency Response Team; ^2^Food and Drug Administration, Silver Spring, Maryland.

Persons with moderate to severe immunocompromising conditions are at risk for severe COVID-19, and their immune response to COVID-19 vaccination might not be as robust as the response in persons who are not immunocompromised* (*1*). The Advisory Committee on Immunization Practices (ACIP) recommends that immunocompromised persons aged ≥12 years complete a 3-dose primary mRNA COVID-19 vaccination series followed by a first booster dose (dose 4) ≥3 months after dose 3 and a second booster dose (dose 5) ≥4 months after dose 4.^†^ To characterize the safety of first booster doses among immunocompromised persons aged ≥12 years during January 12, 2022–March 28, 2022, CDC reviewed adverse events and health impact assessments reported to v-safe and the Vaccine Adverse Event Reporting System (VAERS) during the week after receipt of an mRNA COVID-19 first booster dose. V-safe is a voluntary smartphone-based safety surveillance system for adverse events after COVID-19 vaccination. VAERS is a passive surveillance system for all vaccine-associated adverse events co-managed by CDC and the Food and Drug Administration (FDA). A fourth mRNA dose reported to v-safe or VAERS during January 12, 2022–March 28, 2022, was presumed to be an mRNA COVID-19 vaccine booster dose administered to an immunocompromised person because no other population was authorized to receive a fourth dose during that period (*2*,*3*). In the United States, during January 12, 2022–March 28, 2022, approximately 518,113 persons aged ≥12 years received a fourth dose. Among 4,015 v-safe registrants who received a fourth dose, local and systemic reactions were less frequently reported than were those following dose 3 of their primary series. VAERS received 145 reports after fourth doses; 128 (88.3%) were nonserious and 17 (11.7%) were serious. Health care providers, immunocompromised persons, and parents of immunocompromised children should be aware that local and systemic reactions are expected after a first booster mRNA COVID-19 vaccine dose, serious adverse events are rare, and safety findings were consistent with those previously described among nonimmunocompromised persons (*4*,*5*).

V-safe is a voluntary, smartphone–based U.S. active safety surveillance system established by CDC to monitor adverse events after COVID-19 vaccination (https://vsafe.cdc.gov/en/). The v-safe platform allows registrants to report receipt of a COVID-19 booster dose; new registrants enter information about all doses received. Coincident with authorization for a booster dose in persons with moderate-to-severe immunocompromising conditions, v-safe was updated to allow registrants to enter information about a fourth dose. Registrants aged ≤15 years are enrolled by a parent or guardian. Health surveys sent daily during the first week after administration of each dose include questions about local injection site and systemic reactions and health impacts.^§^ CDC’s v-safe call center contacts registrants who indicate that medical care was sought after vaccination and encourages completion of a VAERS report, if indicated.

VAERS is a U.S. national passive safety surveillance system that monitors adverse events after vaccination and is managed by CDC and FDA (*6*). VAERS accepts reports from health care providers, vaccine manufacturers, and the general public.^¶^ VAERS reports of hospitalization, prolongation of hospitalization, life-threatening illness, permanent disability, congenital anomaly or birth defect, or death are classified as serious.** VAERS staff members assign Medical Dictionary for Regulatory Activities (MedDRA) preferred terms to the findings included in VAERS reports.^††^Serious reports to VAERS were reviewed by CDC and FDA physicians to form a consensus clinical impression. For reports of death, death certificates and autopsy reports are requested and reviewed by CDC physicians to form an impression about cause of death. Reports of myocarditis and pericarditis, rare adverse events that have been associated with mRNA-based COVID-19 vaccines, were identified by searching for selected MedDRA preferred terms; CDC staff members attempted to collect information about clinical course and determined whether the case definition was met.^§§^

In v-safe, a fourth mRNA dose administered ≥3 months after dose 3 to a registrant aged ≥12 years during January 12, 2022–March 28, 2022 (data processed April 17, 2022) was presumed to be an mRNA COVID-19 vaccine booster dose administered to an immunocompromised person; this cutoff date was chosen to reduce overlap with fourth doses administered as a second booster to adults aged ≥50 years, which was recommended on March 29, 2022. The odds of reporting an adverse reaction or health impact after a fourth versus a third dose were compared using a multivariable generalized estimated equations model that accounted for demographic variables, vaccine manufacturer, and repeated measures; p-values <0.05 were considered statistically significant. VAERS adverse event reports after a fourth dose among immunocompromised persons were described by seriousness classification (serious versus nonserious), demographic characteristics, and MedDRA preferred terms; a report of a fourth mRNA dose administered to a person aged ≥12 years during January 12, 2022–March 28, 2022 (data processed April 17, 2022) that did not include MedDRA preferred terms for vaccine error was presumed to be an mRNA COVID-19 vaccine booster dose administered to an immunocompromised person. SAS software (version 9.4; SAS Institute) was used to conduct all analyses. These surveillance activities were reviewed by CDC and conducted consistent with applicable federal law and CDC policy.^¶¶^

## Review of v-safe Data

Overall, 4,015 v-safe registrants reported receiving a fourth dose during January 12, 2022–March 28, 2022, and were presumed to be immunocompromised persons receiving a booster dose; 2,194 persons (54.6%) received mRNA-1273 (Moderna) vaccine and 1,821 (45.4%) BNT162b2 (Pfizer- BioNTech) vaccine. The median registrant age was 62 years (range = 12–94 years); 2,489 (62.0%) were female. In the week after vaccination, local injection site reactions and sys­temic reactions were reported by 1,605 (73.2%) and 1,470 (67.0%) Moderna vaccine recipients, respectively, and by 1,209 (66.4%) and 1,155 (63.4%) Pfizer-BioNTech vaccine recipients, respectively (Table 1). The most frequently reported adverse reactions after dose 4 of either vaccine were injection site pain, fatigue, headache, and myalgia. Local injection site reactions were less frequently reported after dose 4 (70.1%) than after dose 3 (81.7%) (p<0.001); systemic reactions also were less frequently reported after dose 4 (65.4%) than after dose 3 (76.8%) (p<0.001) (Figure).

**TABLE 1:** Adverse reactions and health impacts reported* to v-safe after receipt of a presumed mRNA COVID-19 vaccine booster**^†^** dose among immunocompromised persons (N = 4,015) — United States, January 12–March 28, 2022

Event	No. (%) reporting reaction or health impact after receipt of presumed booster dose		
	Moderna (n = 2,194)	Pfizer-BioNTech (n = 1,821)	Total (N = 4,015)
Any local injection site reaction	1,605 (73.2)	1,209 (66.4)	2,814 (70.1)
Pain	1,512 (68.9)	1,157 (63.5)	**2,669 (66.5)**
Swelling	534 (24.3)	293 (16.1)	**827 (20.6)**
Redness	369 (16.8)	197 (10.8)	**566 (14.1)**
Itching	254 (11.6)	155 (8.5)	**409 (10.2)**
**Any systemic reaction**	1,470 (67.0)	1,155 (63.4)	**2,625 (65.4)**
Fatigue	1,164 (53.1)	884 (48.5)	**2,048 (51.0)**
Headache	863 (39.3)	676 (37.1)	**1,539 (38.3)**
Myalgia	833 (38.0)	659 (36.2)	**1,492 (37.2)**
Joint pain	544 (24.8)	425 (23.3)	**969 (24.1)**
Fever	504 (23.0)	371 (20.4)	**875 (21.8)**
Chills	501 (22.8)	325 (17.8)	**826 (20.6)**
Nausea	295 (13.4)	248 (13.6)	**543 (13.5)**
Diarrhea	152 (6.9)	141 (7.7)	**293 (7.3)**
Abdominal pain	126 (5.7)	127 (7.0)	**253 (6.3)**
Rash	40 (1.8)	35 (1.9)	**75 (1.9)**
Vomiting	21 (1.0)	29 (1.6)	**50 (1.2)**
**Any health impact**	608 (27.7)	455 (25.0)	**1,063 (26.5)**
Unable to perform normal daily activities	543 (24.7)	395 (21.7)	**938 (23.4)**
Unable to attend work or school	203 (9.3)	165 (9.1)	**368 (9.2)**
Received medical care	39 (1.8)	34 (1.9)	**73 (1.8)**
Telehealth	20 (0.9)	14 (0.8)	**34 (0.8)**
Clinic	8 (0.4)	11 (0.6)	**19 (0.5)**
Emergency visit	4 (0.2)	8 (0.4)	**12 (0.3)**
Hospitalization	1 (0.05)	1 (0.1)	**2 (0.05)**

* Percentage of registrants who reported a reaction or health impact ≥1 during days 0–7 after vaccination.

^†^ A fourth mRNA dose ≥3 months after dose 3 administered to a participant aged ≥12 years was presumed to be an mRNA COVID-19 vaccine booster dose administered to an immunocompromised person. Registrants aged ≤15 years must be enrolled by a parent or guardian.

**FIGURE:**
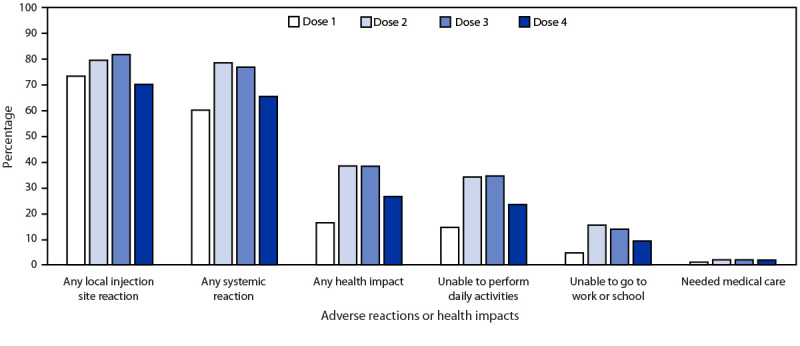
Adverse reactions and health impacts reported to v-safe* after receipt of COVID-19 vaccine doses among persons with presumed immunocompromised status^†^ (N = 4,015), by vaccine dose — United States, January 12–March 28, 2022 * The odds of reporting an event following dose 3 and booster dose were compared for registrants who completed at least one v-safe health check-in survey on days 0–7 after booster dose and ≥1 other dose using a multivariable generalized estimating equations model. This model adjusted for demographic variables and vaccine manufacturer and accounted for repeated measures among doses reported by each registrant (“unable to go to work or school” and “needed medical care” were not adjusted because of small numbers). P-values <0.05 were considered statistically significant. All dose 3 and booster dose differences were statistically significant (p<0.001) except “needed medical care.” † A fourth mRNA dose ≥3 months after dose 3 administered to a registrant aged ≥12 years was presumed to be an mRNA COVID-19 vaccine booster dose administered to an immunocompromised person. Registrants aged ≤15 years must be enrolled by a parent or guardian.

In the week after dose 4 vaccination, 24.7% of Moderna vaccine recipients and 21.7% of Pfizer-BioNTech vaccine recipients reported they were unable to complete their daily activities, and approximately 9% of registrants reported they were unable to attend work or school (Table 1). Fewer than 2% of registrants reported receipt of medical care during the week after dose 4; most who did require care received it through a telehealth appointment. Two registrants reported receiving care at a hospital during the week after dose 4 vaccination. The v-safe call center contacted both these registrants; one completed a VAERS report, and the other indicated the report was accidental or unrelated to vaccination. Inability to work or attend school was less frequently reported after dose 4 (9.2%) than after dose 3 (13.8%) (p<0.001); inability to perform daily activities was less frequently reported after dose 4 (23.4%) than after dose 3 (34.5%) (p<0.001) (Figure). Receipt of medical care was rarely and similarly reported after receipt of either dose 4 (1.8%) or dose 3 (1.9%) (p = 0.70).

## Review of VAERS Data

VAERS received 421 reports from persons who received a fourth dose during January 12–March 28, 2022; 276 (65.6%) of these reports listed a vaccine error. Among reports noting a vaccine error, 225 (81.5%) indicated that no adverse health event occurred.

The remaining 145 (34.4%) reports were presumed to be for immunocompromised persons who received a fourth dose. Among these, 105 (72.4%) reports were for events among females, and the median age was 62 years. Most reports were for nonserious events (128; 88.3%) (Table 2); the nonserious events most commonly reported included headache (30; 23.4%), fatigue (26; 20.3%), and pain (22; 17.2%). One nonserious, preliminary report of myocarditis remains under review. There were 17 (11.7%) reports of serious adverse events. One report of death was received from a manufacturer regarding a patient with pulmonary fibrosis who developed respiratory failure; at the time of publication, no further information was available, and follow-up continues.

**TABLE 2:** Reports to the Vaccine Adverse Event Reporting System of nonserious and serious events after receipt of a presumed mRNA COVID-19 vaccine booster* dose among immunocompromised persons (N = 145) — United States, January 12–March 28, 2022

Reported event	No. (%) reporting
**Nonserious VAERS reports**	**128 (100)**
**Symptom, sign, diagnostic result, or condition (MedDRA PT^†^)**	
Headache	30 (23.4)
Fatigue	26 (20.3)
Pain	22 (17.2)
Fever	18 (14.1)
Chills	15 (11.7)
Dizziness	12 (9.4)
Nausea	11 (8.6)
Rash	9 (7.0)
Conditional aggravated	8 (6.3)
Diarrhea	8 (6.3)
Injection site pain	8 (6.3)
Myalgia	8 (6.3)
Arthralgia	7 (5.5)
Erythema	7 (5.5)
Pain in extremity	7 (5.5)
**Serious VAERS reports** ^§,¶^	17 (100)
**Clinical impression**	
Acute myocardial infarction	1 (5.9)
Anaphylactic reaction	1 (5.9)
Congestive heart failure	1 (5.9)
Chronic obstructive pulmonary disease exacerbation	1 (5.9)
Cerebrovascular accident	1 (5.9)
Diabetic ketoacidosis	1 (5.9)
Disseminated herpes zoster	1 (5.9)
Elevated liver enzymes, vomiting and diarrhea, fever, and arthralgia	1 (5.9)
Heart palpitations	1 (5.9)
Hyperglycemia; burning sensation in upper limb	1 (5.9)
No adverse event reported; vaccine received during mental health hospitalization	1 (5.9)
Pulmonary alveolar hemorrhage	1 (5.9)
Pulmonary embolism	1 (5.9)
Respiratory failure resulting in death in patient with pulmonary fibrosis	1 (5.9)
Respiratory syncytial virus pneumonia	1 (5.9)
Septic shock	1 (5.9)
Urosepsis	1 (5.9)

**Abbreviations:** MedDRA PT = Medical Dictionary for Regulatory Activities preferred term; VAERS = Vaccine Adverse Event Reporting System.

* A fourth mRNA dose not administered in error among persons aged ≥12 years during January 12, 2022–March 28, 2022, was presumed to be an mRNA COVID-19 vaccine booster dose administered to an immunocompromised person. Reports indicating a vaccine error (276) were omitted from this analysis in an attempt to only include fourth doses administered as booster doses to immunocompromised persons.

^†^ Signs and symptoms in VAERS reports are assigned MedDRA PTs by VAERS staff members. Each VAERS report might be assigned one or more MedDRA PTs, which can include normal diagnostic findings. A MedDRA PT does not indicate a medically confirmed diagnosis.

^§^ VAERS reports are classified as serious if any of the following are reported: hospitalization, prolongation of hospitalization, life-threatening illness, permanent disability, congenital anomaly or birth defect, or death.

^¶^ Serious reports to VAERS were reviewed by Food and Drug Administration and CDC physicians to form a consensus clinical impression based on available data.

## Discussion

This report presents safety findings from v-safe and VAERS after receipt of COVID-19 vaccine booster doses during a period when a fourth mRNA dose was recommended only for persons with immunocompromising conditions. Reports to v-safe and VAERS after mRNA booster vaccination among persons who received a fourth dose were similar to previous reports that assessed safety data after dose 3 mRNA booster vaccination among nonimmunocompromised persons (*4*,*5*).

Local and systemic reactions and health impacts were less frequently reported to v-safe after receipt of dose 4 than after dose 3 of the primary series among persons with presumed immunocompromise. Similarly, in previous analyses, among all v-safe registrants aged ≥18 years who received a homologous mRNA booster, systemic reactions were less frequent after dose 3 vaccination than after dose 2 (*5*). Among adolescents aged 12–17 years who received a homologous Pfizer-BioNTech third dose, reactions were reported to v-safe with equal or slightly higher frequency after receipt of that booster dose than after dose 2 (*4*).

Most reports to VAERS related to booster doses among persons with presumed immunocompromise were nonserious; the most common adverse events reported were similar to those reported by persons aged ≥18 years after an mRNA booster (*5*). Serious reports to VAERS among persons with presumed immunocompromise included a range of adverse events; no unusual or unexpected reporting patterns were detected.

The findings in this report are subject to at least six limitations. First, v-safe is a voluntary program; therefore, data might not be representative of the vaccinated U.S. population. Second, it is possible that vaccinees who experience an adverse event could be more likely to respond to v-safe surveys and the reported prevalence of adverse events might overestimate the actual prevalence. Third, as a passive surveillance system, VAERS is subject to reporting biases and underreporting, especially of nonserious events (*6*). Fourth, v-safe does not collect information on immunocompromise, and VAERS does not ask about immunocompromising health conditions; therefore, it is not possible to confirm that vaccine recipients included in this analysis were immunocompromised. Fifth, this report did not examine pattern of reporting by heterologous and homologous vaccination. Finally, a report to v-safe or VAERS alone cannot be used to assess causality.

ACIP recommends that moderately or severely immunocompromised persons aged ≥12 years receive a first booster dose ≥3 months after completion of a 3-dose primary COVID-19 vaccination series and a second booster dose ≥4 months after the first booster. Preliminary safety findings for booster doses among persons with presumed immunocompromise are similar to those among nonimmunocompromised persons; reactions are reported less frequently after booster vaccination than after the last dose of a primary series. It is important that health care providers, immunocompromised persons, and parents of immunocompromised children be advised that local and systemic reactions are expected after a booster dose, and that serious adverse events are rare. CDC and FDA will continue to monitor vaccine safety and will provide updates as needed to guide COVID-19 vaccination recommendations.

**Summary**

**What is already known about this topic?**

Additional doses of COVID-19 vaccine are recommended for immunocompromised persons, and 518,113 fourth doses were presumed administered to this population during January–March, 2022.

**What is added by this report?**

Among presumed immunocompromised persons aged ≥12 years, local and systemic reactions were less frequently reported to v-safe after mRNA booster (dose 4) than after primary series dose 3. Only 17 serious adverse events were reported to VAERS.

**What are the implications for public health practice?**

Serious adverse events after mRNA booster (dose 4) are rare. Immunocompromised persons aged ≥12 years should receive a first booster ≥3 months after a 3-dose primary COVID-19 vaccination series and a second booster ≥4 months after the first booster.

## Footnotes

*
https://doi.org/10.1101/2021.07.08.21259776

†
https://www.cdc.gov/vaccines/covid-19/clinical-considerations/covid-19-vaccines-us.html

§ Health surveys are sent for the most recent dose entered via text messages that link to web-based surveys on days 0–7 after receipt of a vaccine dose; then weekly through 6 weeks after vaccination; and then at 3, 6, and 12 months after vaccination. Local injection site reactions include itching, pain, redness, and swelling. Systemic reactions include abdominal pain, myalgia, chills, diarrhea, fatigue, fever, headache, joint pain, nausea, rash, and vomiting. Health impacts include inability to perform normal daily activities, inability to work or attend school, and receipt of medical care.

¶ Health care providers are encouraged by CDC and FDA to report adverse events to VAERS and are required by COVID-19 vaccine Emergency Use Authorizations to report certain adverse events after vaccination to VAERS, including death. https://vaers.hhs.gov/faq.html

** VAERS reports are classified as serious based on the Code of Federal Regulations Title 21. https://www.accessdata.fda.gov/scripts/cdrh/cfdocs/cfcfr/cfrsearch.cfm?fr

†† Each VAERS report might be assigned at least one MedDRA preferred term. A MedDRA-coded event does not indicate a medically confirmed diagnosis. https://www.meddra.org/how-to-use/basics/hierarchy

§§ In VAERS, acute myocarditis was defined as presence of new onset or worsening of one or more of the following signs or symptoms: chest pain, pressure, discomfort, dyspnea, shortness of breath, pain with breathing, palpitations, or syncope; or two or more of the following signs or symptoms in children aged ≤11 years: irritability, vomiting, poor feeding, tachypnea, or lethargy; and one or more new finding of elevated troponin, electrocardiogram findings consistent with myocarditis, abnormal cardiac function or wall motion on echocardiogram, cardiac magnetic resonance imaging findings consistent with myocarditis, or histopathologic findings consistent with myocarditis; and no other identifiable cause for these findings.

¶¶ 45 C.F.R. part 46, 21 C.F.R. part 56; 42 U.S.C. Sect. 241(d); 5 U.S.C. Sect. 552a; 44 U.S.C. Sect. 3501 et seq.
